# An ecological and embodied approach for training the racecar driver

**DOI:** 10.3389/fspor.2024.1415406

**Published:** 2024-05-30

**Authors:** Gal Ziv

**Affiliations:** ^1^ Motor Behavior Laboratory, The Levinsky-Wingate Academic College, Netanya, Israel; ^2^ Institute of Sport Science and Innovations, Lithuanian Sports University, Kaunas, Lithuania

**Keywords:** ecological approach, embodied cognition, racecar driving, motor racing, sports training

## Abstract

In the dynamic sport of racecar driving, split-second decisions and rapid execution are imperative. Such an environment requires a tight functional coupling of perception and action. This paper introduces an approach for training racecar drivers rooted in ecological and embodied perspectives. It discusses three pivotal affordances of racecar driving: turn-ability, overtake-ability, and defend-ability. The paper also discusses the relevant environment and equipment (i.e., simulators) that can be useful for training racecar drivers. In addition, practice activities relevant for the actual racetrack or to the simulator are discussed. Coaches are encouraged to try and implement the proposed training strategies (or parts of it), evaluating their impact on racing performance. Furthermore, researchers can continue exploring these principles, fostering a fusion of empirical insights with practical expertise from coaches and racing communities. By synergizing empirical research with insights from practitioners, we can refine the strategies employed in the training of racecar drivers.

## 1 Introduction

Racecar driving is characterized by its fast-paced and dynamic nature, necessitating driver-athletes to quickly formulate decisions and execute them within the confines of exceedingly stringent temporal and spatial constraints. Additionally, motor races attract an extensive viewership. For example, during the 2021 season, Formula One races attracted 2.69 million spectators, with three races attracting over 300,000 spectators and 11 races attracting over 100,000 spectators (1). Complementing the live attendance, the television viewership for the same season reached 1.55 billion individuals (1). The enthusiasm for engaging in motor racing is equally noteworthy. In the UK alone, the year 2018 saw a participation of 30,100 competitors across 4,400 racing events (2). Given the substantial interest in spectating and actively partaking in motor racing, an exploration of viable training principles tailored for racecar drivers is of interest.

In a previous paper (3), the author discussed the value of adopting ecological and embodied cognition approaches to understanding racecar driving. In short, the ecological approach underscores the inseparability of individuals from their surroundings (4). In the context of racecar driving, it is important to examine the drivers' performance in tight coupling with elements in their environment, such as the racecar, the racetrack, and the weather conditions. Specifically, the ecological approach suggests that organisms perceive *affordances*—opportunities for action based on the functional coupling of the individual organism and their environment (5). For example, a racecar driver can identify an opening on the track, presenting an opportunity to pass another vehicle. Indeed, the affordance-based control of visually guided action suggests that the primary purpose of our perception is to realize what affordances are available to us (e.g., overtake on the outer or inner edge of the racetrack) and select the most suitable one to support the achievement of our goal (6). From this perspective, skill acquisition is the establishment of a functional fit between the individual and the environment (7).

Supplementing the tight functional coupling between an individual and their environment, which culminates in the perception of affordance, is the embodied cognition perspective. Wilson and Foglia (8) suggested that: “*Cognition is embodied when it is deeply dependent upon features of the physical body of an agent, that is, when aspects of the agent's body beyond the brain play a significant causal or physically constitutive role in cognitive processing*” (p. 1). They added that there are three roles the body (in which cognition is embedded in) can play: *body as a constraint—*in which, depending on the characteristics of the body, certain cognitions will come naturally while others will be more difficult; *body as a distributor*—in which cognition is not limited within the skull but rather distributed to non-neural parts of the body and even to the environment; and *body as a regulator—*in which the body regulates space and time to assure cognition-action coordination (8). From this perspective, and especially from the view of *body as a distributor*, the racecar driver and the racecar should be treated or merged into one unit and cognition is distributed beyond the body to the racecar. Dant (9) suggested that “*The driver-car is neither a thing nor a person; it is an assembled social being that takes on properties of both and cannot exist without both*” (p. 74). This notion traces its origins back even further to the insights of Merleau-Ponty (10) who suggested that “*If I am in the habit of driving a car, I enter a narrow opening and see that I can ‘get through’ without comparing the width of the opening with that of the wings, just as I go through a doorway without checking the width of the doorway against that of my body.*” (p. 165). Thus, the relationship between the driver-racecar unit and the environment emerges as a focal point in racecar driving. Drivers must perceive the relevant affordances of that driver-racecar unit rather than merely considering themselves in isolation.

### 1.1 The main affordances in racecar driving

The affordances available to racecar drivers arise from the constraints imposed on them (3). Newell (11) outlined three categories of these constraints: organismic/individual constraints, environmental constraints, and task constraints. These constraints and the interaction among them establish the boundaries that lead to perceived affordances. Thus, identifying and manipulating these constraints can help coaches design training environments to improve their athletes' performance (12). In racecar driving, individual constraints encompass factors intrinsic to the driver (e.g., reaction time, attention). However, considering the driver-racecar unit as the organism, as required when considering the principles of embodied cognition, introduces further constraints (e.g., tire grip, turn radius, acceleration) (3). The environmental constraints include the weather conditions (e.g., the impact of wind on the car's aerodynamics, ambient temperature variations) and track conditions (e.g., ranging from dry to wet, variations in tarmac temperature). Finally, the task constraints include the racing regulations (e.g., allowing adequate space for other cars on the track, refraining from forcing opponents off the track, and not weaving on a straight).

These constraints interact to establish the boundaries for the three main high-order affordances that the racecar driver should perceive and act upon: Turn-ability, Overtake-ability, and Defend-ability. Turn-ability refers to the driver's capacity to navigate corners on the racetrack at the fastest speed possible (by maintaining the optimal racing line). Overtake-ability refers to the driver's ability to overtake another driver on track. Finally, defend-ability refers to the driver's ability to prevent overtaking attempts of other drivers. However, understanding the necessary actions to attain these relatively abstract affordances can be challenging. For example, how does one execute overtake-ability when different actions can achieve it? Does an opening for overtaking exist on the track's outer edge? Or is the opportunity situated on the inner side? Does it necessitate delayed braking before the turn?

An effective approach to understanding the three high-order affordances is using nested affordances in a means-end hierarchy—a framework that describes goal-oriented systems with three levels: *why, what,* and *how* (13). The “*why*” is the highest level and represents the goal to be achieved (e.g., overtake another car; overtake-ability affordance). This level does not detail the actions that one needs to perform to achieve it. The intermediate “*what*” level represents more specific behaviors enabling the attainment of the overarching goal at the *why* level. Finally, the “*how*” level describes the actions required to execute the behaviors in the “*what*” level (14). To illustrate, consider overtake-ability as the affordance at the “*why*” level—this is the ultimate goal. At the “*what*” level, numerous nested affordances may contribute to achieving this overarching goal. These include navigating the racecar through a gap on the outside of the track or employing early braking to enhance speed upon exiting a turn. Finally, at the “*how*” level, the race drivers' use of the steering wheel, throttle (i.e., gas pedal), brake pedal, and an array of switches and knobs on the steering wheel may allow them to achieve the affordances at the “*what*” level (e.g., swiftly applying the brakes upon sighting the 100-meter marker before a turn, drive through inside of the turn, get on the throttle early, engage battery to optimize engine power).

The purpose of this paper is to provide a training approach for motor racing coaches based on the fundamental principles of the abovementioned ecological and embodied approaches. Furthermore, this training approach has the potential to become a starting point for research endeavors that aspire to refine our understanding of performance and learning in motor racing and improve the training we offer prospective racecar drivers. The following sections will discuss the training of each of the three high-order affordances, but first, a discussion on representative task design, and the racecar driving training environment and equipment is warranted.

## 2 Representative learning design

The importance of representative experimental design was discussed by Brunswik (15) and emphasizes the importance of maintaining the organism-environment relationship encountered in real life during the experiment. Building upon this concept, the term *representative learning design* was more recently suggested (16)—advocating the use, during training, of organism-environment interactions that represent those that the trainees will encounter in real life (e.g., in competition, in a race). It is then imperative for coaches to remember that training activities should aim to represent the demands of the race (17). Indeed, training in a high-fidelity simulator for a multitude of possible scenarios on the racetrack follows the concept of representative learning design.

When devising a training protocol, it is essential to consider the duration of the race and the possible onset of drivers' fatigue. For example, a typical Formula One race extends for approximately 90 min. Throughout this duration, drivers must sustain their attention while enduring a substantial physiological load. Any lapse in driving precision due to lack of concentration or fatigue, whether transpiring on the initial lap or manifesting at the final turn moments before crossing the finish line, holds the potential to result in non-completion of the race. Coaches responsible for racecar drivers' preparation should integrate these race-specific conditions into their training design. This does not dictate that training should universally mirror the entirety of a race (17). Indeed, a comprehensive replication of the entire race might not be practically feasible or needed. Nevertheless, training when physically or cognitively fatigued could prove beneficial if administered in thoughtful and moderate doses.

## 3 Environment and equipment

The importance of embodied cognition in sports has been emphasized previously (18–20). In the case of racecar driving, treating the driver's cognition as embodied in the racecar (and embedded in the environment) is essential because the perception of affordances on the racetrack is based on the driver-racecar unit. It is the driver-racecar unit that will have to overtake another driver-car or defend against overtaking by another driver-car.

To this end, the training apparatus must facilitate a sense of embodiment for the drivers. The best approach to achieving such an embodiment is to train in an actual racecar on a real racetrack. However, this is often unfeasible due to financial implications, the imperative of preserving essential car components (e.g., gearbox, power unit), and the sport's regulations (in Formula 1 for example, a typical race weekend includes only three 1-h practice sessions before the qualifying session and the race itself). Thus, practice time in the actual racecar for a given track is limited. This is where simulators come into the picture, offering a viable alternative.

Although a racecar driving simulator will only partially represent the actual racecar on track, we should strive to allow the driver to feel embodied in the racecar and experience a sense of presence. But what is a simulator? The notion of a simulator entails an endeavor to replicate specific elements of an environment while intentionally omitting others (such as the expenses or dangers of operating in the real environment). Consequently, there are inherent differences between the simulator and the simulated (21). Theoretically, if the sensory stimulation in the simulator (the optic array, other energy arrays) were precisely like those an individual perceived in the real world, there would be no perceptual way for an individual to know whether they are in the real world or the simulator. Such a state can be defined as *stimulus fidelity* (21). As Stoffregen et al. (21) suggested, achieving an absolute stimulus fidelity remains unattainable regardless of technological advances due to at least some inevitable differences in the energy arrays between the simulator and the real world. Thus, our task centers on devising a simulation system that offers an experience that mimics the functional essence of real-world experience.

So, what must racecar driving simulators include? First, precise replication of the seat, steering wheel, brake pedal, and accelerator pedal are required. This means, for example, that the knobs and buttons embedded in the actual steering wheel should also be present in the simulator's steering wheel. Furthermore, the resistance and movement of the pedals should match those in the real racecar. Second, the algorithms that determine tire degradation, grip levels in varying environmental conditions (e.g., dry/wet tarmac, cold/hot tarmac, wind conditions), aerodynamic behavior, and general car behavior should, as much as possible, match the real racecar on a real racetrack. Third, the energy arrays should mimic those experienced on the racetrack. These include the optical flow, the auditory flow, and the haptic flow. Maintaining these simulator characteristics are in line with the concepts of representative learning design (16).

Of all the above mentioned demands, the haptic flow is the most difficult to simulate. It is unlikely, for example, that the driving simulator will precisely simulate the actual g-forces the driver experiences or the exact feeling when the car is on the verge of losing grip. However, some of those sensations could be delivered by haptic feedback to the steering wheel. Moreover, advanced simulators offer full motion and attempt to mimic the g-forces that act on the driver's body (for example, by using pneumatic pressure that can be delivered through the seat and seatbelts). These advanced technologies seem promising, but they do not represent the actual sensation in the racecar. Still, highly realistic optic and auditory flow simulation, with moderately realistic haptic flow simulation, might be enough to provide a positive transfer of learning from the simulator to the racetrack.

Finally, as mentioned earlier, the simulator should give the driver a sense of embodiment. This can be somewhat subjective, and developing this sense will depend on continuous discussion with the driver about how it can be improved (see Table 1 for a list of requirements from a racecar simulator). All the above mentioned requirements are responsible for creating a successful training simulator, but the variables that make up the functional fidelity are most important as constraints for simulator design (21).

**Table 1:** The four basic requirements of a racecar driving simulator.

Requirement	Description
Functional fidelity/racecar behavior	Algorithms of grip, aerodynamics, and car behavior in all environmental conditions are similar to the actual car. Driver's actions in the simulator have the same effects as they would in the actual racecar
Structural fidelity	The car seat, steering wheel including knobs and buttons, gas and brake pedals, should match the actual racecar
Energy arrays	Optical and auditory arrays should match those on the actual racetrack; haptic sensations through the steering wheel, seat, and seatbelts, if possible
Embodiment	The driver should have a subjective sense of being embodied in the car and embedded in the virtual environment

## 4 Training for turn-ability

Turn-ability can be defined as skillfully navigating a racecar through a corner at the maximum achievable speed while maintaining a grip on the track's surface. Accomplishing this demands an artful mastery of steering, braking, and accelerating. In addition, the turn-ability affordance is closely related to the ability of the driver to identify, choose, and follow the optimal racing line—the path that allows maintaining the highest possible speed around the track. In fact, this is a crucial aspect of racecar driving.

Several constraints interact and affect the ability of the driver to turn the car at high speeds and maintain the optimal racing line. These factors include, among other things, tire conditions, weather conditions, and the car's aerodynamic profile (3). Furthermore, a driver's ability to rapidly process information at high speeds is important. Drivers must accurately identify when to brake as they approach a corner, determine the apex of the turn, and ascertain the optimal moment to start accelerating. Certain variables that affect a driver's decisions change from one lap to the next, such as the diminishing grip caused by tire degradation (3), changes in brake temperatures, car damage, etc.

All the above mentioned factors create a dynamic situation in which performance is constrained by interacting variables, some of which change constantly. Under such conditions, perceiving turn-ability requires constant perceptual attunement during the whole race, as each lap introduces a sequence of corners, often distinct from one another, necessitating varying inputs to be applied to the steering wheel and pedals. This improved attunement would lead to better (re)calibration^1^ to the changing variables and thus, to better performance.

The drivers' ability to perform these turns successfully can be seen as a problem-solving activity (22). Indeed, Myszka et al. (22) suggested that athletes face continually changing information. On the racetrack, the environment-car-driver relationship changes continually, leading to a different set of constraints faced by the driver as they reach the same corner in different laps.

In this respect, Fajen (23) showed that information in the optical array is sufficient for participants to recalibrate their braking performance after, unbeknownst to them, braking force decreased. In that study, participants were asked to perform a braking task to stop at a target. Feedback was eliminated one second after the onset of braking; thus, only information in the optical flow was available. Without feedback about the final position error relative to the target and without knowing that the brake force changed, participants rapidly recalibrated to the decrease in the braking force.

Similarly, racecar drivers need to recalibrate their actions to the changing variables in what can be defined as a continuously controlled visually guided action [see (6, 23) for an in-depth discussion on continuous visually guided actions]. However, the information required to perform such a task is not only visually guided but also relies on auditory and haptic information. For two main reasons, drivers cannot rely on feedback to navigate the racecar through a corner at the maximum possible speed successfully. First, in such a fast-paced race-driving scenario, feedback for performed actions arrives too late. For example, suppose drivers perceive they have enough grip to accelerate rapidly as they exit the corner. If this perception proves inaccurate, they will have to react to an unexpected loss of grip. This could result in losing valuable lap time in the best-case scenario or losing control of the car in the worst-case scenario. Second, due to dynamic changes in the car's properties from lap to lap, feedback from a previous lap may not be relevant to the current lap. Hence, race drivers must recalibrate their actions based on attunement to perceptual information.

Training drivers to achieve turn-ability relies on challenging them with various tracks, types of corners, track conditions, car conditions, and individual conditions (e.g., physiological and/or cognitive fatigue). For this purpose, coaches should set up different scenarios in the simulator where the driver is required to achieve the fastest lap times under a variety of the abovementioned conditions. Coaches should encourage drivers to find an optimal solution among the available landscape of affordances. For example, when training to reach the fastest lap time, as expected in a qualifying session of a race, drivers should train with and without cars on track. Ideally, a “flying lap,” which refers to a measured lap during a qualifying session, is conducted without the presence of any other cars obstructing the path. However, other cars are often on the track when a driver tries to achieve the fastest lap time. The drivers of these other cars are supposed to stay off the optimal racing line and may be penalized if they fail to do so. Still, the driver on the flying lap should practice with those cars on the track as they may present different constraints, especially when reaching a corner. The more drivers practice with different scenarios, the better they can recalibrate in the face of such disturbances. This, in turn, will enable them to attain an optimal lap time or, at the very least, reduce the risk of losing substantial fractions of a second during their lap. Similarly, drivers should attempt to achieve the fastest lap times with somewhat used tires,^2^ different car setups, etc.

A final noteworthy aspect concerning the perception of affordances is their reliance on an individual's capacity for action. As these capabilities diminish, certain affordances that were once feasible might become unattainable. Furthermore, once alterations occur in one's action capabilities, a corresponding shift must also occur in the perception of affordances (6). But what are the action capabilities in racecar driving? This can be examined from the point of view of the driver or the driver-racecar unit. Throughout a race, drivers encounter a high physiological load (24, 25) that can affect their capacity to sustain attention and operate the steering wheel and the pedals over the race's duration. But assuming coaches have prepared the driver-athletes well, and they are fit to operate the race car, the focal point should shift to examining the driver-racecar unit as the reference for action capabilities. It is these capabilities that change, and recalibration is dependent on. It is the degradation of the tires, the availability of extra power from the battery, the damage the car may have sustained that has affected aerodynamics, the weight of the racecar as fuel is being consumed, etc. Each of these factors relates to the driver-racecar unit and will affect the ability to perceive and act upon the nested affordances leading to the overarching turn-ability affordance.

In summary, training for turn-ability in racecar driving requires that drivers encounter many probable scenarios they are likely to face on the racetrack. The more drivers face such scenarios, the better they will be able to improve their perception of the affordances and (re)calibrate their actions to achieve the fastest lap times.

## 5 Training for overtake-ability

Previous research has demonstrated that drivers of passenger cars base their overtaking maneuvers on perceiving overtake-ability (26, 27). However, in scenarios where cautious and law-abiding driving practices are upheld, these overtaking maneuvers occur within relaxed spatial and temporal constraints. In motor racing, overtaking is naturally a critical factor. Racecar drivers are required to perceive optimal overtaking opportunities, but unlike regular passenger car drivers, this often unfolds within tight temporal and spatial constraints. In addition, due to the dynamic nature of the sport, there are numerous possible scenarios of overtaking based on many interacting variables, such as the condition of both the overtaking car and the car being overtaken (e.g., tire conditions), the relative positions of cars on the track and their respective velocities, and the understanding of intentions and capabilities of other drivers on track—and thus the affordances available to them. This intricate web of factors generates nearly limitless permutations of overtaking possibilities within the racing environment and thus the drivers face a difficult problem-solving scenario.

In such conditions, the ability to make the correct decision—to overtake or not to overtake—can be challenging. On one hand, drivers can “play it safe” and refrain from overtaking even though it may have been possible. While this strategy enhances their prospects of completing the race, it diminishes their likelihood of securing a victory. On the other hand, drivers can adopt a more daring tactic by attempting to overtake whenever they perceive the slightest chance of success. Such a strategy will increase their chances of winning a race but will decrease their chances of finishing it because the likelihood of making a race-ending error rises. This balancing act of knowing when to execute an overtake and when to drop back and wait for a better chance is at the heart of motor racing. The question that emerges is how drivers can be effectively trained to enhance their capacity to arrive at the correct decision amidst such complex conditions.

Seifert et al. (28) suggested that maintaining metastability in a dynamic movement system can be useful in developing expertise. Metastability is a property of dynamic movement systems that combines movement flexibility and behavioral consistency (28). Kelso (29) suggested that metastability is “*the simultaneous realization of two competing tendencies: the tendency of the components to couple together and the tendency for the components to express their intrinsic independent behavior*” (p. 186). Practically, this means that there are no clear behavioral attractors when one is in a metastable state. Instead, there is an attractiveness toward different attractors, and thus, different behavioral possibilities can be pursued [see (30) for an in-depth discussion of flexibility and attraction toward consistent behaviors]. For example, Hristovski et al. (31) showed that when boxers were at a scaled distance (physical distance divided by their arm length) of 0.6 from a punching bag, they presented the most varied choices of punches. This represents a metastable region that allows for flexibility in movement selection. As the authors of this study suggested, training in the regions of this scaled distance can be optimal for practicing various punches in novice boxers.

Similarly, the motorsport coach can create simulated overtaking scenarios, strategically situating the prospective race driver within a metastable realm wherein numerous potential actions are viable. As drivers confront these conditions, they must perceive the optimal affordances that lead to their overarching goal of overtaking. This corresponds well with the idea of a rich landscape of affordances (32) taken from ecological psychology. Deliberately crafting scenarios that position race drivers within metastable situations empowers them to perceive and choose from an array of plausible (nested) affordances, compelling them to make decisive selections to identify the most advantageous one. Such practice will likely improve drivers' perceptual attunement, consequently leading to improved performance.

To put the theoretical concepts above in practical terms, an example can be helpful.^3^ It can be argued that certain overtaking scenarios provide no more than one optimal and easily discernible affordance. Consider a scenario where a driver trails another by 300 msec on a long enough straight with a 10–15 k·hr^−1^ closing speed. In such a scenario, executing the overtake presents minimal challenge, as the driver is positioned to complete the maneuver effortlessly. Under such conditions, it is unlikely that the driver will have to choose between competing affordances given that the optimal affordance is obvious. Similarly, suppose the driver trails another by 750 msec or more, and the closing speed is less than 5 k·hr^−1^ on a shorter straight. In this scenario, the decision to postpone the overtake in anticipation of a better overtaking opportunity later is the only likely alternative. Again, there are no competing affordances to choose from.

**Figure 1:**
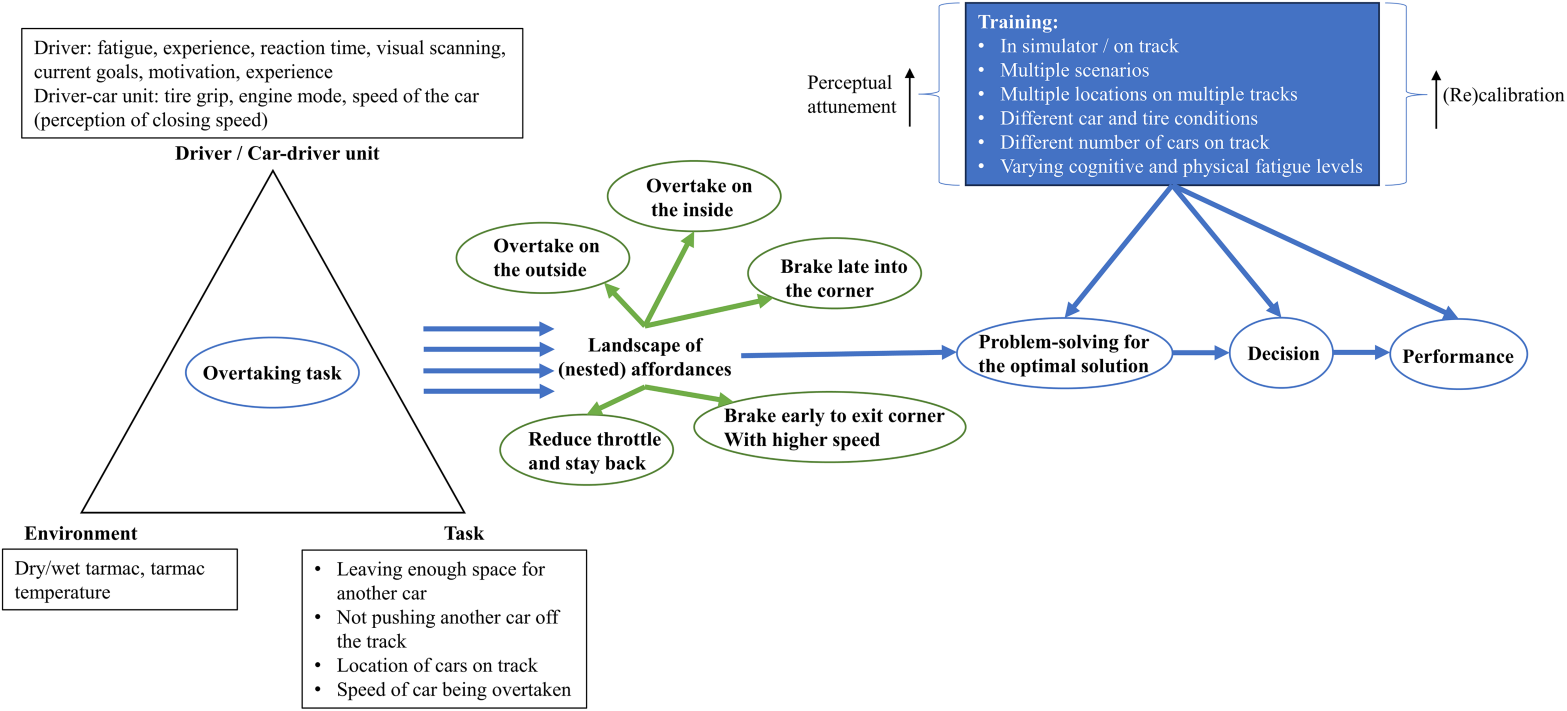
An analysis of an overtaking task. The driver must solve the problem of finding the optimal (nested) affordance based on the perception of the constraints. Then, the driver should reach a decision and act upon it. Training can improve perceptual attunement and the ability to (re)calibrate actions that will lead to a successful overtake.

However, when the driver's position lies within 300–750 msec behind another driver, with a closing speed of ∼5–10 k·hr^−1^, and with a medium-length straight, a scenario materializes in which the driver is confronted with a spectrum of competing affordances (see Figure 1). The driver can decide to: (1) try to brake late and execute the overtake, (2) choose an earlier braking point and try to overtake by maximizing speed when exiting the corner, (3) attempt to overtake on the outer edge of the track, (4) attempt to overtake on the inner edge of the track, or (5) ease off the throttle and avoid the overtaking attempt. In addition, coaches should also vary the constraints in such scenarios to include different environmental conditions (wind, temperature), tarmac conditions (dry/wet, temperature), and tire conditions (degradation, temperature).

Using a variety of constraints across a range of scenarios that put the driver in a metastable region can lead to practice conditions that will allow drivers to improve their perceptual attunement to the available information. Improved attunement will lead, in turn, to improved decision-making and performance in varied scenarios that can be encountered in a race. In contrast, confining training to scenarios characterized by a singular feasible affordance holds limited efficacy, particularly once the driver is proficient in maneuvering the car. Coaches should also bear in mind the importance of familiarizing the driver with a variety of race start conditions (e.g., position on the grid at the start, distance to first corner). These instances present unique scenarios in which all cars are in close proximity, and the windows of opportunity for overtaking emerge and vanish within mere fractions of a second.

In summary, the role of the coach is to develop training scenarios that require the racecar driver to choose between various potential affordances. Such training is meant to improve drivers' perceptual attunement and thus, the ability to find the optimal affordance within the landscape of affordances. Conversely, engaging in training exercises centered around straightforward scenarios wherein a single unmistakable affordance prevails is improbable to yield substantial improvements in the drivers' performance once they have acquired the requisite motor skills to address that specific affordance adeptly.

## 6 Training for defend-ability

It has been previously shown that individuals can perceive the affordances available to other people (33, 34). Fajen et al. (35) added that perceiving the affordances of opponents is of significance in sports. If athletes know the action possibilities of their opponents, they can: (1) choose how to act to improve their chances of success and (2) adopt actions designed to entice the opponent into choosing a less-than-optimal course of action, thereby establishing what can be termed as an “*affordance trap*” (3). These “*affordance traps*” are rooted in the notion that affordances are not only opportunities for behavior, but can also be invitations for behavior (36).

In motor races, drivers are required to defend against being overtaken. To do so, they should be aware of the potential affordances available to the drivers that try to overtake them. Defend-ability is constrained by several factors. First, regulatory bodies institute rules that comprise the task constraints. Such task constraints can include leaving enough space on the track for the other racecar and avoiding weaving on a straight. These rules allow for fair and safe racing. Additional constraints are related to the condition of the car and the tires, which determine the existing level of grip, the conditions of the tarmac (wet/dry, temperature), the speed of both racecars, the nature of the upcoming corner (fast/slow, sharp/hairpin/chicane), as well as the weather and visibility conditions. The interaction between those constraints will influence the drivers' choice of one of a few possible actions, such as: blocking the inner or outer sections of the track, breaking late before the turn, or adopting an early breaking strategy before the turn to allow the other car to overtake only to regain position by accelerating more swiftly upon exiting the corner. Similarly to overtaking scenarios, the interplay of interacting constraints yields an array of potential scenarios that racecar drivers may encounter as they attempt to defend their position on the racetrack.

To improve drivers' ability to defend against overtaking attempts, coaches should prioritize the driver's ability to perceive the affordances available to the overtaking driver. Analogous to the training suggestions for overtake-ability, coaches should expose drivers to a range of scenarios in which they must defend their position on the racetrack. Coaches and drivers should be aware that much of the information about the actions and intentions of other drivers can be gathered from regularly checking the mirrors. This acquired information will enable the driver to perceive the affordances available to the other driver. Consequently, the driver can make informed decisions, such as whether to secure the inner or outer edges of the track or opt for late or early braking.

Furthermore, by perceiving the overtaking affordances accessible to other drivers, defending drivers can establish an “Affordance trap” by selecting various racing lines and braking points. For example, a driver might decide to brake early on the outside of the corner, luring the overtaking driver to brake late on the inside of the corner, only to emerge from the corner with greater velocity and reclaim the position. Finally, the option of not defending can also be strategically viable. This decision arises when the odds of thwarting the overtake are slim (e.g., when the defending driver is operating with significantly degraded tires), and any defense maneuver would merely result in a loss of valuable lap time.

In summary, effectively defending a track position requires that drivers perceive the affordances available to other drivers as they try to overtake them. Based on the available information, drivers can choose from various affordances that may lead to a successful defense or realize that defend-ability is not currently possible.

## 7 Coaching on the racetrack and in the simulator

For race drivers, practice for enhancing racing skills can occur either in the simulator or on the actual racetrack. However, practice time on the racetrack is limited. As mentioned earlier, in a typical Formula 1 race weekend, for example, there are only three 1-h practice sessions available. Moreover, practice opportunities on the racetrack are limited to select few activities. These include experimenting with different car setups, identifying optimal braking points, turn-in points, adapting to tire degradation during turns, and (re)calibrating to the car's behavior with varying fuel loads. However, practice sessions on the racetrack do not allow practicing skills related to overtaking or defensive driving, nor do they accommodate practicing race starts. Therefore, to improve these crucial skills, drivers must utilize simulation training.

In this respect, coaches can structure practice sessions around the concept of *alive movement problems*—problems of varying complexities, often unpredictable, that athletes face as they step on the playing field (37). Importantly, *alive movement problems* encompass the emergence and disappearance of affordances as environmental cues continually evolve. While this concept was introduced mainly in invasion sports (37), Myszka et al. (22) proposed its relevance to various other sports as well.

Racecar driving coaches can leverage this concept by designing practice activities that prepare drivers to devise optimal integrated movement solutions (22) for such *alive movement problems* encountered during a race.

Therefore, on-track practice sessions should prioritize tasks related to turn-ability, particularly focusing on experimentation to identify the optimal racing line. In contrast to the actual racetrack, the simulator provides an ideal environment for coaches to create diverse overtaking and defensive scenarios that mirror real-world challenges. This allows drivers to enhance their ability to formulate integrated movement solutions for such scenarios, thereby refining their racecraft skills effectively. Table 2 presents examples of practice activities for the racetrack and for the simulator [also see Table 1 in (22), for a checklist on designing practice for solving *alive movement problems*]. Choosing the correct practice activities to the practice modality (racetrack or simulator) can increase efficiency of practice.

**Table 2:** Examples of practice activities suitable for the racetrack and for the simulator.

On the racetrack	
Goal	Specific Examples
Find the optimal racing line around each bend.	Experiment with: Braking early, braking late, until optimal braking point is found. Experiment with varying fuel loads, types of tires, and stages of tire degradation to find how the racing line or braking points change.
Experiment with approaching a corner from inside or outside.	Tweak braking points and turn-in points as if there are other cars on track that force to take a corner not on the racing line.
In the simulator	
Practice: Overtaking affordances Defending affordances	Experiment with: Different number of cars, different distances between cars, different locations on track, etc. Allow and encourage creative overtaking/defending solutions. Let the drivers encounter different scenarios randomly—require active problem-solving. Make sure the driver faces quickly emerging and fading overtaking/defending opportunities.
Practice race starts	Experiment with: Late reaction time of driver or late reaction times of drivers in front. Reaching the first corner on the inside or outside Reaching the first corner at the same time as another car. Encourage drivers to try different lines and braking points.

## 8 Summary

In this paper, I tried to provide an approach for training the perceptual-motor skills of racecar drivers from ecological and embodied perspectives. I explained the three primary high-order affordances that racecar drivers should be aware of, alongside a selection of the nested affordances that can allow race drivers to act on these primary affordances successfully. The training approach is rooted in solid theoretical underpinnings but, at the same time, lacks empirical validation. Subsequently, it is up to coaches to consider adopting this approach (or parts of it) and assess its impact on enhancing drivers' performance.

The preliminary nature of this perspective paper makes it apparent that refinement of the suggested training is plausible, predicated upon the accumulation of data gathered from racing clubs that opt to use it. Furthermore, I hope this paper will inspire researchers to study the possible benefits of such training. Combining empirical data from future studies with insights drawn from coaches and racing clubs can help develop and refine the training we provide to racecar drivers.

## Data Availability

The original contributions presented in the study are included in the article/Supplementary Material, further inquiries can be directed to the corresponding author.

## Footnotes

^1^(re)calibration refers to the process of correctly mapping the detected information to actions. Once (re)calibration is achieved, performers know how to stay within their action capabilities (Fajen, 2007a).

^2^In certain cases, the racing team can decide to save fresh tires for the race itself.

^3^Note that the values in this example are somewhat arbitrary and are only presented here to make a point. The actual values can change greatly between different types of racecars, races, and track conditions.
